# Bridging the molecular and clinical aspects of resveratrol in Alzheimer’s disease: a review

**DOI:** 10.1007/s13205-025-04451-x

**Published:** 2025-08-06

**Authors:** Liju Susan Mathew, Aradhana Marathe, Akash Aman, Anmol Vats, Teresa Joy, Y. Lakshmisha Rao

**Affiliations:** 1 Department of Biomedical Sciences, College of Medicine, Gulf Medical University, Ajman, United Arab Emirates; 2 Department of Biochemistry, Kasturba Medical College Mangalore, Manipal Academy of Higher Education, Manipal, India; 3 Kasturba Medical College Mangalore, Manipal Academy of Higher Education, Manipal, India; 4 Department of Anatomy and Medical Imaging, American University of Antigua College of Medicine, Antigua, WI USA; 5 Department of Anatomy, Kasturba Medical College Mangalore, Manipal Academy of Higher Education, Manipal, India

**Keywords:** Alzheimer’s disease, Neurodegeneration, Resveratrol, Neuroprotective, Anti-inflammatory, Anti-oxidant

## Abstract

Alzheimer’s disease (AD) is a progressive brain disorder that affects neurological functioning specifically targeting cognition, memory and behaviour. Pathology starts with the accumulation of tau proteins which on phosphorylation can be destructive for brain function. With gold standard drugs like Donepezil, there have been several attempts made to discover adjuvant therapeutic molecules such as plant-based products that could not only ease the ill-toward effects of these drugs, but also lead to the betterment of the patients suffering from AD. These are crucial in the management of patients of AD, by associations with psychological vulnerabilities and the overall loss of health of such individuals ‘Resveratrol’ is one such plant-based molecule which is a stilbene polyphenol present in many of the commonly occurring plants. Resveratrol is reported to have anti-oxidant and anti-inflammatory properties which brings about the neuroprotection. Several studies have also been conducted that targets the signalling cascades involved during the progression of AD. This review attempts to give a collective information of its properties, its synthesis, metabolism and mechanisms that could drive researchers forward for its therapeutic applications during the treatment of Alzheimer’s disease.

## Introduction

Man is considered superior when it comes to his intellect and use of wisdom. The brain development, structure, function, its sympathetic and para-sympathetic co-ordination makes him ‘Strongest of all’. However, what if, this investiture of cognition gets affected? What can be done to prevent it or correct it?

Epigenetic factors such as stress, pollution, drugs, apoptosis, aging, or antibody-related pathologies can lead to fundamental change in structure–function relation of the brain. (Kubota 2016) Alzheimer’s Disease (AD) is one such progressive brain disorder that primarily starts affecting the memory, cognition and finally impacting behaviour (Zvěřová 2019). According to recent research data reveals that, by 2050, the prevalence of the AD will be doubled and Europe and tripled in all over the world.AD is considered as most devastating and challenging disorder since its complicated pathophysiology (Scheltens et al. 2021). The pathophysiology of AD includes, intra-neuronal accumulation of hyper-phosphorylated tau protein in the form of neurofibrillary tangle, and deposition of extra cellular beta amyloid plaques. In addition, the oxidative stress is the major cause for these pathophysiology (Huang et al. 2016, Zheng and Wang 2025). Structurally, a normal tau is an unfolded, highly soluble protein, and is accumulated in neuronal axons and dendrites (Mietelska-Porowska et al. 2014). These tau proteins, collaborating with the microtubules, play a major role in maintenance of the neuronal structure and neuronal transport (Pradeepkiran and Reddy 2019). However, it is also observed that, phosphorylated form of tau proteins become cytotoxic. Thus phosphorylation state of tau protein determines the normal functioning of a tau protein. The hyper activated phosphatases will lead to formation of paired helical filament and neurofibrillary tangle reducing the stability of microtubules leading to their disruption (Yu et al. 2018). Several studies propose oxidative stress as a pathogenic mechanism in AD. In AD, the evidence for oxidative stress in brain are increased iron, alteration in protective enzymes and markers of oxidative damage in proteins and lipids. Another major pathological feature of the AD brain is senile plaques. These plaques are microscopic lesions seen in the parenchyma of the brain and are featured by abnormal accumulation of proteins, added with dystrophic neuronal processes and reactive glial cells. Though the senile plaques are seen in aged brain, presence of numerous such plaques was a diagnostic feature of AD (Walker 2020; Nelson et al. 2012). The principal protein in the core of the plaque was explained for the first time by Glenner and Wong in 1980s.They described a partial amino acid sequence of the protein in cerebral amyloid angiopathy (CAA), from patients with Down’s syndrome and AD. Later Masters and Beyreuther also found the same protein as the key component of plaques. Initially these proteins were named as the β protein, A4 or β/A4, which were termed as Aβ in later days. Recently this Aβ proteins are popularly named as Aβ plaques (Masters et al. 2006, Benson et al. 2018). Amyloid plaques are insoluble peptide clumps, formed by the abnormal cleavage of amyloid precursor proteins (APP). Normally these APP are cleaved by the enzymes called, *γ* secretase, *β* secretase and α secretase. However, AD brain, the cleavage of APP takes place in an abnormal site by a variant *γ* secretase. In addition, this abnormal cleavage leads to production of non-soluble aggregation of 42 amino acid peptide named, Aβ 42 or Aβ, which is popularly termed as *β* amyloid plaques. The *β* amyloid plaques play a major role in pathogenesis of AD and popularly called as “amyloid cascade hypothesis”. This Accumulation of *β* amyloid plaques is known to form neurofibrillary tangles in the brain, further causing vascular damage and neuronal death resulting in dementia (Medeiros 2011). Many naturally occurring nutraceuticals have also been promising in the treatment AD. Resveratrol is one such polyphenolic compound (Ma 2014). AD is a known progressive neurodegenerative disorder with complex pathophysiology. The pharmacological therapies for AD which are currently in use, aim at its symptoms, such as memory loss and cognitive impairment. The therapeutic strategies used in treatment of AD include inhibition of the acetylcholine esterase, and acetylcholine synapse maintenance. Usually Choline esterase inhibitors such as Donepezil, Rivastigmine, Galantamine, and NMDA blockers like Memantine are used as gold standard drugs to manage AD with or/and antipsychotics modalities. (Yiannopoulou 2020). It is observed that, these drugs have failed to show their efficacy in treating the AD, certainly there is a significant research gap in improving the therapeutic modules. Since oxidative stress is a major pathophysiology in AD, antioxidant components could be an effective therapeutic agent. Previous studies have shown the efficacy of polyphenol compounds in providing antioxidant support in human brain. Resveratrol is a naturally occurring polyphenolic compound and has gained attention in pharmacology due to its antioxidant, anti-inflammatory, anti-cancerous and anti-aging properties. This review aims at collating the research evidence and research gap on resveratrol.

### Biosynthesis of resveratrol in higher plants

Resveratrol, (chemically C_14_H_12_O_3_: 3, 5, 4′-trihydroxy-*trans*-stilbene) a natural stilbenoid phenol or polyphenol, and a phytoalexin produced by several plants. In higher plants, its biosynthesis is activated by stress factors, such as UV radiation, infection, and mechanical wounding (Meng et al. 2023, Rao et al. 2020). Stilbene synthase (STS) and chalcone synthase (CHS), the key enzymes in flavonoid biosynthesis, initiate polyketide formation, leading to aromatic ring generation via a Claisen-like reaction. STS influences the final product of this metabolic pathway. The process progresses through a series of reactions, including decarboxylation, dehydration, and enolization of stilbene-2-carboxylic acid, producing the active resveratrol (Rivière et al. 2012, Dewick 2001; Rupprich et al. 1978).

### Metabolism of resveratrol

Primarily, resveratrol is metabolized through Phase II enzyme biotransformation pathways through the liver Cytochrome P_450_, which occur in the cellular mitochondria to increase water solubility by attaching a polar moiety (Phang and Lierena 2025). The purpose of Phase II metabolism is to form stable, water-soluble products and less toxic metabolites that can be excreted by the body. Thus, catabolized Resveratrol is further absorbed in the small intestine by enterocytes, where it undergoes glucuronidation to form large polar metabolites, which are then excreted renally as inactive compounds (Kuhnle et al. 2000, Qiu et al. 2017).

The high metabolism (Gambini et al. 2015) and naturally low concentration of resveratrol in food sources reduce its oral bioavailability. This factor raises concerns about its pharmacokinetics and the ability of biological active concentrations to reach body tissues, particularly in brain tissue (Cottart et al. 2010). The administration of trans-resveratrol-loaded lipid-core nano capsules and tissue distribution studies preserve effective bioavailability in an experimental rat brain (Frozza et al. 2010). Evidence-based nutritional studies have shown that a resveratrol treatment of 500 mg/day results in plasma concentrations of only approximately 10–71.2 ng/ml in healthy human volunteers, which can be attributed to its poor bioavailability (Sergides et al. 2016). With the same dosage, the plasma concentrations of resveratrol metabolites vary: the glucuronidated form reaches 4083.9 ± 1704.4 ng/ml, the sulfated form 1516.0 ± 639.0 ng/ml, while the unmetabolized form remains at 71.2 ± 42.4 ng/ml (Sergides et al. 2016).

### Studies on dosage of resveratrol

The range of Resveratrol doses in animal models is 0.1 mg/kg to 1000 mg/kg body weight. Mice treated with low doses of RS (5 mg/kg/day) have gained weight, but mice treated with large doses (400 mg/kg/day) have lost weight. According to Johnson et al., rats given a high dose of Resveratrol exhibited hepatomegaly, but there was no histological proof of hepatotoxicity (Johnson 2011). According to Chow et al.'s human study, it may be the source of negative side effects in females, including diarrhea, heartburn, increased hunger, mood swings, and menstrual irregularities (Chow 2010). Human investigations have suggested that a dose of 1gm/day of Resveratrol is well-tolerated (Johnson 2011, Goldberg 2003). Since it is an antioxidant, taking it orally is recommended safe.

### Role of resveratrol in AD

Pathology of AD involves hippocampus, a region of high neuroplasticity considered by ongoing myelination and neurogenesis in adulthood (Ma et al. 2014; Jurkowski et al. 2020; Marzola et al. 2023). It is most evident that, various aspects of neuroplasticity take place at the structural level of adult brain, including dendritic arborization, axonal sprouting, synaptic remodelling, synaptogenesis, along with neurogenesis (Esiri and Chase 2012). Resveratrol has been shown to enhance hippocampal plasticity, improve cognition, and positively influence mood (Dias et al. 2016). The neuroprotective role of resveratrol in AD involves multiple mechanisms, with key findings and potential impact towards disease progression is listed in Table 1.

**Table 1:** Role of resveratrol in Alzheimer's disease involving mechanism, key findings, impact and previous observers

Mechanism	Key findings	Impact	Previous observers
Aβ and Tau pathology reduction	Biomarker reductions in CSF Aβ40, Aβ42, and serum Aβ40 in AD patients	Decline in AD-related biomarkers	Kocatürk et al. (2022)
	Decreases Aβ42 and p-tau in SAMP8 mice	Reduces AD markers in mouse models	Farr et al. (2013); Chen et al. (2018)
	Significant decline in Aβ counts and burden in RV-fed mice	Reduces Aβ aggregation and burden	Chen et al. (2019)
	Lowers hyperphosphorylated tau levels in AD mice	Reduces tau pathology	
	Reverses SIRT1 inactivation and tau hyperphosphorylation in rats and mice	Modulates SIRT1 to reduce tau pathology	Azargoonjahromi and Abutalebian (2024)
Mitochondrial function and bioenergetics	Enhances mitochondrial biogenesis, improves synaptic function, upregulates mitochondrial antioxidant enzymes, and decreases ROS	Reduces oxidative stress and supports neuronal haemostatics	Singh and Singh (2025)
	Reduced mitochondrial complex IV activity leads to ROS accumulation	Accelerates AD progression	Misrani et al. (2021)
Resveratrol and cellular pathways	Reduced SOD levels contribute to oxidative stress in AD	High-dose resveratrol enhances SOD activity	Houldsworth (2024); Bartra et al. (2024)
	SOD2 overexpression prevents memory deficits	Reduces hippocampal superoxide levels	Massaad et al. (2009)
	Resveratrol inhibits NADPH oxidase, preventing apoptosis in brain endothelial cells	Protects against glucose-induced apoptosis	Chen et al. (2013)
	FDG-PET shows reduced glucose metabolism in early AD	Indicates glucose utilization decline	Cozza et al. (2023)
	Microglial activation triggers cytokine release via NLRP3 inflammasome	Promotes Aβ plaque formation	Spangenberg and Green (2017)
	Microglial activation enhances synapse loss and worsens tauopathy	Contributes to neurodegeneration	Leyns et al. (2017)
	Reduced BH4 levels impair monoaminergic neurotransmission	Increases neurodegenerative disease risk	Fanet et al. (2021)
	Increasing TTR levels stabilizes the tetramer, enhancing Aβ binding	Prevents Aβ aggregation	Santos et al. (2016)
Resveratrol and autophagy	Post-mortem AD brains show increased autophagosomes	Leads to abnormal Aβ accumulation	Martínez et al. (2023)
	Resveratrol promotes autophagy	Supports cellular homeostasis, neuroprotection	González-Reyes et al. (2017)
Resveratrol and AD protection	Resveratrol reduces Aβ toxicity, prevents BBB impairment	Inhibits Aβ1–42 crossing into the brain	Zhao et al. (2015)
	Aβ deposition in microvascular endothelial cells enhances APP production	Positive Aβ-CAA in large vessels leading to vascular damage, impaired blood blow and cognitive decline	Cozza et al. 2023
	Sirtuins (SIRT1, SIRT3, SIRT6) regulate mitochondrial function and Aβ metabolism	SIRT1, SIRT3 linked to Aβ accumulation	Ruankham et al. (2021); Pukhalskaia et al. (2020)
Resveratrol and neuroprotection	Resveratrol modulates SIRT1 in the hippocampus	Protects against Aβ1–42-induced learning, memory deficits	Wang et al. (2017) Wang et al. (2018b)
	Resveratrol inhibits NF-κB, modulates MAPK, ERK1/2, and PI3K/Akt pathways	Reduces oxidative stress via Nrf2 activation	Zhou et al. (2022)

The neuroprotective properties of resveratrol have shown to decrease the Aβ peptides aggregation, in the hippocampus and there by improve memory and cognitive function by promoting ongoing neurogenesis (Kempermann et al. 2015; Biscaro, et al.^2012^; Chance et al. 2011; Thomas and Smith 2013; Frozza et al. 2013). This brain modulating metabolite is usually found in grapevines, grape juice, wine, peanuts, pomegranates, spinach, jackfruit, and bananas (Popović et al. 2017; Singh et al. 2016; Beckeret al. 2017; Tripatthi et al. 2023). The earliest history of resveratrol extraction dates to 1939 from the roots of the Japanese plant *Polygonum cuspidatum* (Timmers et al. 2012). This therapeutic compound has been extensively used in traditional medicine for over two thousand years due to its multifaceted properties, including anticancer, platelet anti-aggregation, antioxidant, anti-aging, anti-frailty, anti-inflammatory, and antiallergenic activities (Gambini et al. 2015).

### The neuroprotective and antioxidant role of resveratrol

Evidence have shown that resveratrol triggers mitochondrial biogenesis both in vitro and in vivo by upregulating mitochondria-localized antioxidant enzymes, thereby reducing the production of reactive oxygen species (ROS) (Oliveira et al. 2017; Jardim et al. 2018; Rao et al. 2024). This established multifaceted mechanism has shown that AMPK, the cellular energy sensor, promotes mitochondrial biogenesis and inhibits anabolic processes.

**Fig. 1:**
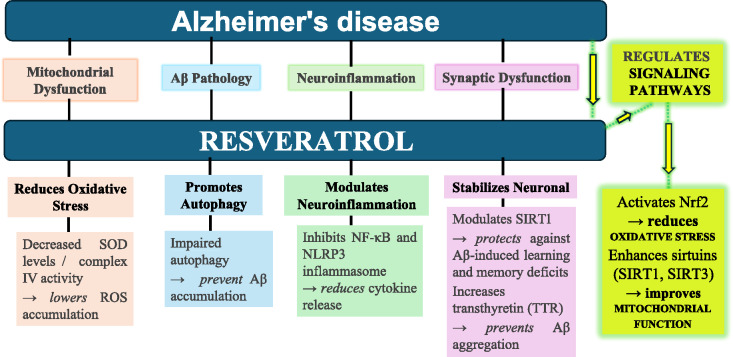
Schematic representation of resveratrol and its neuroprotective effect against Alzheimer's disease progression through the regulatory signaling pathway

The role of mitochondrial ROS in microglial activation remains unclear. However, evidence suggests that mitochondrial activity is modulated by resveratrol through the SIRT1 functional pathway, which in turn prevents MAPK and NF-κB activation in microglial cells. In addition, interaction with RanGap1, a cytoplasmic GTPase, contributes to downstream signalling triggered by ROS. SIRT1 has also been shown to regulate mitochondrial function and influence intracellular ROS levels through the resveratrol–SIRT1–RanGap1 axis (Su et al. 2022a; Su et al. 2022b; Shan et al. 2022) (Fig. 1).

**Fig. 2:**
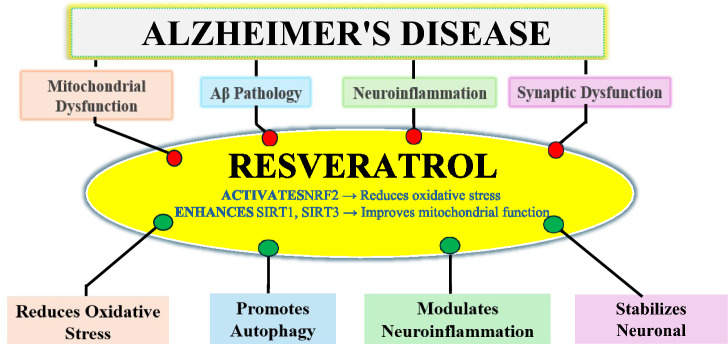
Representation of mechanism of anti-oxidant effect of resveratrol on AD

Beyond the molecular mechanisms at the cellular level, neuronal and glial cells in the grey matter of healthy individuals demand high concentrations of adenosine triphosphate (ATP), as oxygen (O₂) consumption occurring at a significant rate of approximately 3.5 ml of O₂ per 100 g of brain tissue per minute. Mitochondrial dysfunction, particularly reduced mitochondrial complex IV activity, leads to excessive reactive oxygen species (ROS) release and toxicity (Bernier et al. 2011; Rink and Khanna 2011; Misrani et al. 2021), contributing to neuroinflammation and cell death. These disruptions contribute to neurodegeneration and accelerate the progression of Alzheimer's disease (AD), as shown in Fig. 2.

Here, resveratrol has demonstrated to activate neuroprotective mechanisms by eliminating damaged mitochondria, offering protection against Aβ-induced oxidative damage in PC12 cells in an in vitro Alzheimer’s disease model (Wang et al. 2017; Wang et al. 2018a). While the purified extract of (E)-resveratrol, with its complex structure and bioactive components, has been shown to modulate key inflammatory pathways, such as nuclear factor-kappa B (NF-κB) and mitogen-activated protein kinases (MAPKs), thereby inhibiting the production of inflammatory cytokines and chemokines. Simultaneously, resveratrol and SIRT1 activation counteract which effects by promoting mitochondrial biogenesis, reducing oxidative stress, and enhancing synaptic function (Singh S 2024; Rai Singh 2024; Singh and Singh, 2025). Consequently, the cytotoxic, anti-cancer, and neuroprotective effects of resveratrol exhibit dose- and time-dependent properties, decreasing NF-κB activity and FOXO protein-mediated apoptotic activity, respectively (Kim et al. 2012; Sharifi-Rad et al. 2021). When Salehi and his research team examined the antioxidant activity of resveratrol, they observed this property was most distinct in its dose-dependent pro-oxidative effect. With short-term exposure, resveratrol induces oxidative stress, whereas prolonged exposure results in less pronounced cytotoxicity. Consequently, surviving cells become more resistant to resveratrol-induced damage, and its effects diminish over the course of the treatment period (Salehi et al. 2018). Resveratrol, administered at a dose of 20 mg/kg body weight, was evaluated in chemically induced rat models of Alzheimer’s disease, a reduction in oxidative stress biomarkers and improve behavioural performance was reported.

Due to complex nature of the disease profile and growing interest among researchers in AD, the use of natural aging animal models has often been limited, due to high maintenance costs and long experimental cycles. Simultaneously the senescence-accelerated mouse (SAM) model is also not preferred because of its high mortality rate and poor drug absorption. Although Streptozotocin (STZ) -induced AD mice exhibit hyperphosphorylation of tau protein, prolonged exposure has been shown to be carcinogenic to humans. Zhao et al. 2016) used aluminium chloride (AlCl₃), administered intraperitoneally at doses of 4 mg/kg or 40 mg/kg for approximately 40 days; however, in vivo studies were discontinued due to poor bioavailability. Similarly, transgenic models carrying PSEN1 and PSEN2 mutations demonstrate comparable pathological features of AD but are limited by a weak correlation with behavioural changes.

### Reduction of superoxide biosynthesis

Superoxide dismutase (SOD) is an essential antioxidant enzyme belonging to the oxidoreductase family, with a metal cofactor at its catalytic core (Ighodaro and Akinloye 2018). SOD plays a crucial role in innate antioxidative defence and helps slow the progression of AD. The expression of amyloid-beta (Aβ) at the protein level, indicated high-dose resveratrol significantly enhances SOD activity, whereas low-dose resveratrol shows no notable effect, suggesting that only higher doses effectively increase antioxidant capacity in AD models (Kong et al. 2019). Oxidative stress is linked to neuronal damage, as reduced SOD levels are observed in AD (Rao et al. 2021) (Houldsworth 2024; Bhatt et al. 2021). In a mouse model, SOD2 isoenzyme overexpression prevented memory deficits by reducing hippocampal superoxide levels (Massaad et al. 2009; Zhao et al. 2016). Resveratrol have shown evidence of effectively increasing the mitochondrial enzyme, the first-line defence genes SOD2, and chronic intake induces direct antioxidant effects while inhibiting the oxidative damage (Bartra et al. 2024). In the normal adult central nervous system (CNS), SOD1 is expressed in the pyramidal neurons of the hippocampal and cortical regions. Astrocytes, which play a critical role in maintaining cognitive function by providing energy substrates to neurons, responding to injury, and regulating the synaptic environment, also express SOD1. In excitotoxic injury, downregulation of SOD1 in neurons precedes neuronal degeneration (Peluffo et al. 2005). However, SOD1 expression in astrocytes is upregulated in response to injury, while SOD2 expression increases following oxidative stress, aiding in ROS detoxification (Maier et al. 2002; McBean 2018). This additionally highlights a key link between astrocyte redox capacity and the pathological phenotype of astrocytes in aging and AD (Habib et al. 2020).

### Inhibition of ROS production mediated by NADPH oxidase

Resveratrol inhibits NADPH oxidase and high glucose (HG)-induced endothelial cell apoptosis in murine brain microvascular endothelial cells. HG upregulates the NADPH oxidase subunit Nox1 but not Nox2, Nox4, or p22phox expression through NF-κB activation, leading to elevated ROS production (Chen et al. 2013). Evidence also suggests that genes associated with energy metabolism in microglia (in mice) express GLUT1 (SLC2A1) [found in the blood–brain barrier] and GLUT3 (SLC2A3) [found in neurons], support both glycolytic and oxidative energy metabolism. A proteomic study has recognized activated microglial immunometabolism of glucose as a key factor in inflammatory responses during neurodegeneration (Johnson et al. 2020). FDG–PET had shown greater decline in glucose utilization and the posterior part of cingulate cortex is known to be affected metabolically in the early clinical stages of AD (Cozza et al. 2023). Microglial activation triggers the secretion of inflammatory cytokines following NLRP3 inflammasome activation, leading to the release of apoptosis-associated speck-like protein containing a caspase specks, which seed new Aβ plaques. This process can be both neurotoxic by promoting synapse loss (Schindler et al. 2019; Wu et al. 2015; Kapogiannis et al. 2011; Spangenberg and Green 2017) and exacerbating tauopathy-mediated pathology (Leyns and Holtzman 2017; Leyns et al. 2017).

### Tetrahydrobiopterin and neurodegeneration

Decreased peripheral and/or central levels of tetrahydrobiopterin (BH_4_) and its low bioavailability increase the risk of neurodegenerative diseases. BH_4_ oxidation reduces its availability as an enzymatic cofactor, leading to altered monoaminergic neurotransmission (Fanet et al. 2021).

### GTP energy dependence of endocytosis and autophagy

Local GTP levels regulate autophagy, and dysregulated autophagy has been linked to aging and AD. Interestingly, post-mortem analysis of AD brains has revealed the accumulation of autophagosomes and prelysosomal autophagic vacuoles in dystrophic neurites and synaptic terminals. In early AD stages, upregulation of autophagosomes in hippocampal CA1 pyramidal neurons is associated with altered expression of autophagy-related genes (ATG3, ATG5, ATG12, ULK1, and PIK3C3/VPS34) and proteins (LC3B-II and LC3B-I) (Bordi et al. 2016). Alterations in autophagosome trafficking, generate abnormal β-amyloid (Aβ) accumulation, and increased Rab5 activity contributing to enhanced amyloid precursor protein (APP) endocytosis. These metabolic adaptations are linked to the mechanism of seeded polymerization and neurofibrillary tangles and amyloid deposition, with Rab5 expression being upregulated in hippocampal CA1 neurons (Ginsberg et al. 2010; Omar 2017; Martínez et al. 2023).

### Endothelial nitric oxide synthase

Nitric oxide (NO) is a known paracrine regulator of vascular tone, vascular smooth muscle proliferation, platelet aggregation, and leukocyte adhesion, as well as blood pressure regulation. Endothelial nitric oxide synthase (eNOS)-deficient mice exhibit markedly elevated lesion volume and reduced cerebral blood flow (An et al. 2021; Yadav 2017). Evidence suggests that increased Aβ deposition in the brain, enhance amyloid precursor protein generation and Aβ secretion in vitro by activating microvascular endothelial cells (Austin et al. 2010). The cerebral Aβ-amyloid angiopathy (Aβ-CAA) in large vessels is more consistently positive for Aβ40, though Aβ42 and leading to vessel damage, impairing blood flow, and cognitive decline (Cozza et al. 2023).

### Silent information regulator

Sirtuin is a conserved protein, made of nicotine adenine dinucleotide dependent histone deacetylase which regulate, critical signalling pathways. Sirtuin-1 (Sirt1) biologically regulate pathway involved in gene expression, metabolism, and cellular senescence (Cantó et al. 2009). While the antioxidant activity of resveratrol promotes neuronal differentiation through the indirect activation of silent information regulator-1 (SIRT1) (Beher et al. 2009; Rao et al. 2022). SIRT1 plays a crucial role in modifying mitochondrial activity (Su et al. 2022b), regulating neuronal growth and differentiation, and preventing apoptotic cell death by deacetylating and repressing p53 activity. Substantial pathophysiological processes in AD have been shown to highlight Sirt1 role in Aβ and tau metabolism (Mehramiz et al. 2023; Liu et al. 2022). The absence of SIRT1 in hippocampal neurons is linked to cognitive impairment, as SIRT1 deacetylates p53, NF-κB, and FOXO, preventing neuronal apoptosis (Li et al. 2014; Ramis et al. 2015). By inhibiting ROS production through SIRT1 activation, resveratrol prevents Aβ deposition and neurodegeneration, offering neuroprotection against oxidative damage (Wang et al. 2010; Li et al. 2014). Evidence suggests that SIRT1 activation by resveratrol protects against Aβ-induced microglial death and enhances cognitive function. The neuroprotective effects of resveratrol against monomeric C-reactive protein (mCRP) involve modulation of the SIRT1, Nrf2, and NF-κB pathways, leading to reduced production of inflammatory mediators and increased activity of antioxidant enzymes (Bartra et al. 2024). Furthermore, SIRT1 overexpression enhances neuronal protection and regulates antioxidant responses by modulating ROS, nitric oxide (NO), proinflammatory cytokine production, and Aβ expression in the brains of individuals with Alzheimer's disease (Kim et al. 2007). Catenin pathway can also influence the neurogenesis in the embryonic stage and therapeutic approach at this level can be beneficial. (Ramakrishnan 2023; Chakravarthy et al. 2023; Rai et al. 2020).

Evidence shows that Aβ production is regulated by amyloid-β precursor protein (AβPP), BACE1 (β-secretase), and ADAM10 (α-secretase) (Qin et al. 2006). BACE1, the rate-limiting enzyme in Aβ generation, is often overexpressed. Its expression is suppressed when PPARγ and PGC-1α are deacetylated by SIRT1 (Wang et al. 2013). Simultaneously, ADAM10 cleaves AβPP into a soluble, neuroprotective fragment known as sAβPP. Upregulation of ADAM10 promotes non-amyloidogenic processing, reducing Aβ accumulation and enhancing neuroprotection in Alzheimer’s disease (McKenzie et al. 2017). In addition, SIRT1 promotes autophagy in AD by enhancing the deacetylation of Beclin-1 (Rupprich and Kindl 1978).

The expression of SIRT1, SIRT3, and SIRT6 in the saliva of AD was identified to be 1.5–4.9-fold reduced compared to the health senile group SIRT6 was 2.5–4.5-fold lower than in healthy (Pukhalskaia et al. 2020).Experiments on animal models has proved intermittent food deprivation decreases hyperexcitability of neuronal network and augments deficits in hippocampal synaptic plasticity in a SIRT3-dependent manner (Ruankham et al. 2021). In a mouse model, SIRT3 expression follows the pattern and timing of amyloid-beta (Aβ) accumulation, while its higher levels in the temporal neocortex of humans suggest a response to disease progression, potentially as a protective or compensatory mechanism (Esteves et al. 2019).

The inheritance of the ε4 allele in the APOE gene is a significant genetic risk factor associated with Alzheimer's disease. Apolipoprotein E (ApoE) isoforms, particularly ApoE4, interfere with Aβ metabolism, leading to its accumulation. ApoE4 competes with transcription factor EB (TFEB) for coordinated lysosomal expression and regulation sites, impairing the autophagic process in APOE ε4 allele carriers and facilitating AD onset (Parcon et al. 2018). Resveratrol administration for 45 days has been shown to reduce Aβ toxicity in mice, highlighting its chemo-preventive role against plaque formation and oxidative stress (Karuppagounder et al. 2009). In addition, resveratrol improves learning and memory, prevents blood–brain barrier (BBB) impairment, inhibits Aβ1–42 from crossing the BBB, and reduces hippocampal Aβ accumulation (Behar et al. 2009; Zhao et al. 2016). In a mouse model, SIRT3 expression follows the pattern and timing of amyloid-beta (Aβ) accumulation, while its higher levels in the temporal neocortex of humans suggest a response to disease progression, potentially as a protective or compensatory mechanism (Esteves et al. 2019).

### Anti-inflammatory effects and microglial modulation

Resveratrol promotes anti-inflammatory Th2 responses, increasing anti-inflammatory cytokines while suppressing the neurodegenerative activity of M1 microglia (Gomes et al. 2018; Rao et al. 2023). SIRT1 expression regulates p53 and peroxisome proliferator-activated receptor-gamma coactivator 1α (PGC-1α), decreasing Aβ accumulation and improving mitochondrial dysfunction (Sweeney and Song 2016). In animal models, intracerebroventricular injection of resveratrol reduces hippocampal damage and neurodegeneration, with a concurrent decrease in SIRT1 acetylation (Lalla and Donmez et al. 2013; Rege et al. 2014). Similarly, study indicate that resveratrol modulates SIRT1 expression in the rat hippocampus, protecting neurons against Aβ1–42-induced disruption of spatial learning, memory, and synaptic plasticity (Wang et al. 2017). These findings reinforce its therapeutic potential in neurodegenerative conditions like AD by mitigating ischemic damage and plaque-induced toxicity (Marambaud et al. 2005).

### Molecular mechanisms and signalling pathways

Resveratrol as expressed previously, exerts neuroprotective effects through multiple pathways, particularly by inhibiting nuclear factor-κappa B (NF-κB) and modulating key signalling cascades. These include, P38 mitogen-activated protein kinase (P38-MAPK), Extracellular signal-regulated kinase one-half (ERK1/2), Phosphoinositide 3-kinase (PI3K)/Akt pathways. It also reduces oxidative stress by activating diverse signalling pathways, such as Nrf2 and phosphorylated Nrf2 (Zhou et al. 2022; Rai 2016; Prakash 2014). In addition, resveratrol promotes autophagy, which plays a vital role in cellular homeostasis and neuroprotection (González-Reyes et al. 2017; Li et al.^2018^; Sadhukhan et al. 2018; Jia et al. 2017).

In Alzheimer's disease (AD), AMPK activation has been associated with excessive autophagy-mediated secretion of Aβ (Son et al. 2016; Assefa et al. 2020) and direct phosphorylation of tau (Domise et al. 2016). Dysregulation of the metabolic pathway, coupled with loss of mitochondrial function activate AMPK signalling, contributing to the core hallmarks of AD, including Aβ accumulation, tau aggregation, and oxidative stress (Herzig and Shaw 2018), ultimately leading to neurofibrillary tangle formation and Aβ plaque deposition (Caberlotto et al. 2013). Resveratrol-induced activation of AMPK has been shown to reduce ROS levels and extracellular Aβ concentration (Vingtdeux et al. 2010) while also increasing NAD⁺ availability and modulating tau acetylation (Cheng et al. 2014).

Toll-like receptors (TLRs) in AD of the microglia and neurons activate the canonical NF-κB signaling pathway, leading to the expression of proinflammatory factors (Chiarini et al. 2020; Hou et al. 2019). NF-κB inhibitors have been shown to reduce NF-κB-induced APOE activity (Sun et al. 2022). Within this cellular mechanism, the p65 subunit of NF-κB binds to promoter regions, inducing the expression of β-secretase (Chen et al. 2012). Although NF-κB-associated genes may prevent tau hyperphosphorylation and the accumulation of Aβ plaques (Yan et al.^1995^; Sivamaruthi et al. 2023; Prajapati et al. 2025). Simultaneously dysregulation of NF-κB signalling has been shown to increase neurofibrillary tangle formation and Aβ plaque accumulation (Zhan et al. 2018; Yang et al. 2021). Thus SIRT1–AMPK–NF-κB axis, influenced by metabolic and oxidative states; have shown to be vital to the AD pathophysiological. Efforts must focus on understanding the underlying molecular mechanisms to identify targets for prevention and therapeutic intervention.

### Drug interaction and safety of resveratrol

There is no substantial evidence of a medication interaction with RSV, despite the fact that there are several studies (Timmers 2012; Chow 2010; Johnson 2011) regarding the pharmacological advantages of Resveratrol. According to Sahoo et al. (Sahu 2013), Resveratrol can cure the cognitive impairment in rats that have been exposed to prenatal stress. This implies that Resveratrol has neuroprotective properties and is safe for pregnant rats. Resveratrol has been utilized to boost the bioavailability of curcumine and piperine Johnson 2011) and it has demonstrated effectiveness without causing any interactions. However, other phenolic compounds have been shown to have negative effects, thus more research is needed to demonstrate the safety of Resveratrol (Soo et al. 2016). Resveratrol has shown the Synergistic role when used with the gold standard drug Donepezil, whose mechanism though unclear reflects on anti-oxidant and anti-inflammatory pathways that work through microglia and astrocytes. (Rao 2023, 2024).

## Conclusion

Evaluating the effects of resveratrol in AD cases, both in human and animal studies, highlights its potential therapeutic or protective role. However, limitations related to dosage regimens, treatment durations, and outcome measures must be acknowledged. Resveratrol’s indirect role in regulating synaptic plasticity and neurogenesis, which are critical for early diagnosis and prevention of the disease, especially as the aging population is growing more rapidly needs to further studied. Dosage and the long-term effects of use of resveratrol needs to be substantiated. In vitro and in-silico studies could give us a better approach in understanding the mechanistic regulation of Resveratrol as an adjuvant in the pathogenesis and treatment of AD.

## Data Availability

Not Applicable (As this manuscript is a narrative review, this does not include any data).
